# Glycosylation of dentin matrix protein 1 is a novel key element for astrocyte maturation and BBB integrity

**DOI:** 10.1007/s13238-017-0449-8

**Published:** 2017-08-18

**Authors:** Bo Jing, Chunxue Zhang, Xianjun Liu, Liqiang Zhou, Jiping Liu, Yinan Yao, Juehua Yu, Yuteng Weng, Min Pan, Jie Liu, Zuolin Wang, Yao Sun, Yi Eve Sun

**Affiliations:** 1 Tongji University School of Medicine, Stem Cell Translational Research Center, Tongji Hospital, Shanghai, 200065 China; 2 Department of Oral Implantology, School of Stomatology, Tongji University, Shanghai Engineering Research Center of Tooth Restoration and Regeneration, Shanghai, 200072 China; 3 Department of Psychiatry and Biobehavioral Sciences, David Geffen School of Medicine, University of California, Los Angeles, CA 90095 USA; 4 Collaborative Innovation Center for Brain Science, Tongji University, Shanghai, 200092 China

**Keywords:** blood-brain barrier, dentin matrix protein1, glycosylation, astrocyte, cell adhesion, proteoglycan

## Abstract

The blood-brain barrier (BBB) is a tight boundary formed between endothelial cells and astrocytes, which separates and protects brain from most pathogens as well as neural toxins in circulation. However, detailed molecular players involved in formation of BBB are not completely known. Dentin matrix protein 1 (DMP1)-proteoglycan (PG), which is known to be involved in mineralization of bones and dentin, is also expressed in soft tissues including brain with unknown functions. In the present study, we reported that DMP1-PG was expressed in brain astrocytes and enriched in BBB units. The only glycosylation site of DMP1 is serine89 (S89) in the N-terminal domain of the protein in mouse. Mutant mice with DMP1 point mutations changing S89 to glycine (S89G), which completely eradicated glycosylation of the protein, demonstrated severe BBB disruption. Another breed of DMP1 mutant mice, which lacked the C-terminal domain of DMP1, manifested normal BBB function. The polarity of S89G-DMP1 astrocytes was disrupted and cell-cell adhesion was loosened. Through a battery of analyses, we found that DMP1 glycosylation was critically required for astrocyte maturation both *in vitro* and *in vivo*. S89G-DMP1 mutant astrocytes failed to express aquaporin 4 and had reduced laminin and ZO1 expression, which resulted in disruption of BBB. Interestingly, overexpression of wild-type DMP1-PG in mouse brain driven by the nestin promoter elevated laminin and ZO1 expression beyond wild type levels and could effectively resisted intravenous mannitol-induced BBB reversible opening. Taken together, our study not only revealed a novel element, i.e., DMP1-PG, that regulated BBB formation, but also assigned a new function to DMP1-PG.

**Electronic supplementary material:**

The online version of this article (doi:10.1007/s13238-017-0449-8) contains supplementary material, which is available to authorized users.

## INTRODUCTION

The blood-brain barrier (BBB) is a specialized and dynamic system that controls material transport from circulation to brain parenchyma, and protects brain from toxic substances in the blood. BBB allows passages of water, some gases, lipid-soluble molecules, and selective transport of some molecules, while acts as a pathogen-insulating barrier to create an immune privileged microenvironment within the central nervous system (CNS) (Persidsky, et al., 2006; Alvarez, et al., 2011).

Multiple types of cells are well organized in BBB, which include endothelial cells, astrocytes, and pericytes (Zhao et al., 2015; Attwell et al., 2010; Knowland et al., 2014; Armulik et al., 2010; Dalkara and Alarcon-Martinez, 2015). Among these cells, astrocytes are the most abundant cells in the brain, which wrap around endothelial cells by using their endfeet to hermetically-seal the crevice between endothelial cells (Romero et al., 2003; Wolburg and Lippoldt, 2002). The soma of astrocytes extends out thick and short primary processes, which branch extensively in brain parenchyma, forming polarized terminal end feet to encase blood vessels. Perivascular astrocyte end feet, along with targeted endothelial cells of the cerebral blood vasculature, form waste-clearance channels between astrocytes and blood vessels, creating a glymphatic drainage system in brain.

Although BBB is such an important boundary structure of the brain and its disruption is believed to be associated with many neurological disorders, very little is known about the molecular mechanisms regulating the structure and function of BBB. A recent study reveals that astrocytic glycoproteins, laminin-111 and laminin-211 regulate pericyte differentiation and maintain the integrity of BBB (Yao et al., 2014). Another study indicates a novel role for laminin-dystroglycan interactions in mediating integrations between astrocytes and endothelial cells, which participate in regulating the function of BBB (Menezes et al., 2014).

Dentin matrix protein1 (DMP1) is first discovered in a cDNA library of odontoblasts, which turns out to be essential for maturation of odontoblasts and osteoblasts (George et al.,^1993^; George et al.,^1995^). DMP1 is proteolytically processed into two fragments, with the C-terminal being the key fragment for biomineralization, which is absolutely critical for bone development (Feng et al., 2006; Lorenz-Depiereux et al., 2006). Interestingly, the N-terminal of DMP1 is glycosylated to become a chondroitin sulfate enriched proteoglycan, which also positively regulates osteogenesis (Qin et al., 2006; Sun et al., 2015). DMP1 is found to be expressed in non-mineralized tissues as well, including brains and muscles (Terasawa et al., 2004; Rowe et al., 2000). Our preliminary data showed that the major form of DMP1 in the brain was DMP1-proteoglycan. We therefore assumed that glycosylated DMP1 might have unique biological functions in the central nervous system (CNS). In mouse, DMP1 has only one serine glycosylation site (Ser89), which is highly conserved among species and could be modified by chondroitin 4-sulfate glycosaminoglycan (Qin et al., 2006). It is also known that glycosylation of DMP1 can be fully eradicated in HEK-293 cells by the S89G substitution mutation (Peng et al., 2008). In bone tissues of S89G-DMP1 mutant mice, this mutation dramatically reduces glycosylation levels of DMP1, confirming Ser89 is a key glycosylation site (Sun et al., 2015). In addition, we found that expression of DMP1 in brain tissues was enriched in astrocytes within the BBB unit. In this study, we employed S89G-DMP1 mutant mice to uncover the function of DMP1-PG during astrocytes maturation and maintenance of BBB integrity.

## RESULTS

### DMP1 was expressed in BBB units and acted as proteoglycan

**Figure 1:**
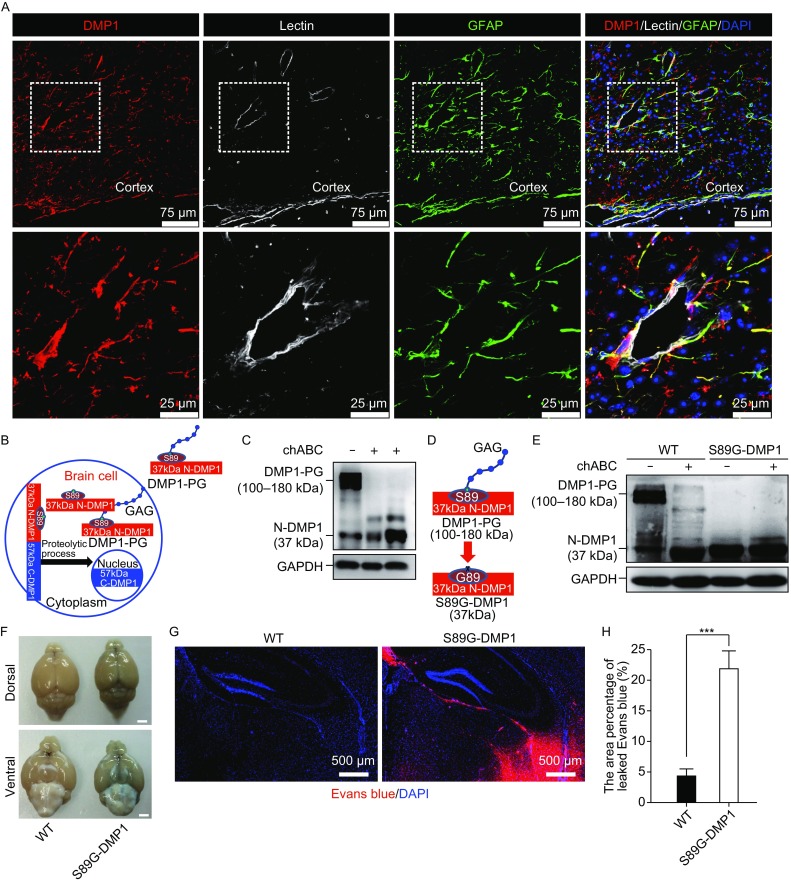
**DMP1 is expressed in BBB units and acts as a proteoglycan to regulate BBB function**. (A) Immunofluorescence images of lectin labeled mouse brain vasculatures, which are wrapped around by astrocytic end feet, positive for GFAP and DMP1. DAPI labels nuclei. (B) A schematic diagram of DMP1 full-length protein, as well as C-DMP1 and N-DMP1 after cleavage. N-DMP1 can be glycosylated on S89. (C) ChondroitinaseABC treatment was used to demonstrate that N-DMP1 was glycosylated; (D) Knock-in mouse strategies to change S89 to glycin, which eliminates N-DMP1 glycosylation; (E) Immunoblot showed that S89G-DMP1 brain was devoid of N-DMP1-PG. (F) Brains dissected from mice injected with Evans blue intraperitoneally; (G) After perfusion, brain sections were used to examine Evans blue directly under fluorescent microscope. (H) Quantification of brain areas leaked with Evans blue. At least 9 random captures from 3–4 mice per genotype were quantified. ***, *P* < 0.001, mean ± SEM

DMP1 is an extracellular matrix (ECM) protein. The expression of DMP1 was detected in mouse brain, especially in the blood-brain barrier unit (Fig. 1A). BBB is composed of GFAP positive astrocyte end feet wrapping around blood vessels, which completely seals blood vessels, insulating brain tissues from circulation. DMP1 was found to co-localize with GFAP rather than lectin in BBB units, even though both GFAP and DMP1 were expressed around lectin, which outlined the vasculature. This observation suggested that DMP1 was more associated with astrocytes than endothelial cells.

DMP1 can be detected in four protein forms: 57 kDa DMP1 C-terminal (C-DMP1), 37 kDa DMP1 N-terminal (N-DMP1), glycosylated N-terminal core protein (N-DMP1-PG, also referred to as DMP1-PG), and a small amount of non-processed full-length DMP1 (Fig. 1B). A high molecular weight of N-DMP1 was detected by Western blot of brain lysate using N-terminal specific antibodies, which could be converted to 37 kDa core N-DMP1 by Chondroitinase ABC (chABC) (Fig. 1C). To reveal the role of glycosylation of DMP1, we generated a point mutation of DMP1 glycosylation site in a mouse model (S89G-DMP1) through homologous recombination (Fig. 1D). In this model, the glycosaminoglycan (GAG) chain of DMP1 was largely removed (Fig. 1E).

### Point mutation of DMP1 glycosylation site led to BBB disruption

To assess the function of BBB in S89G-DMP1 mutant mice, we injected Evans blue intraperitoneally and observed leakage of this dye into the brain parenchyma of mutant mice (Fig. 1F–H). Quantitative analysis showed that, in comparison with wild type controls, the area with Evans blue leakage in brain parenchyma was dramatically increased (*P* ≤ 0.001 vs. WT). This assay indicated that BBB integrity was compromised in S89G-DMP1 mice. Previous studies on dentinogenesis and osteogenesis have shown that C-DMP1 is an important element in regulating bone development and mineralization (George et al., 1995; Feng et al., 2006). We created brain-specific conditional C-DMP1 knockout mice by crossing C-Dmp1^flox/flox^ mice with Nestin-Cre transgenic mice. The brain-specific knockout of C-DMP1, surprisingly, did not lead to BBB disruption (Fig. S1), suggesting that N-DMP1 (rather than C-DMP1) was important for BBB structure and/or function.

### S89G-DMP1 inhibits astrocytes to locate to and wrap around blood vessels

**Figure 2:**
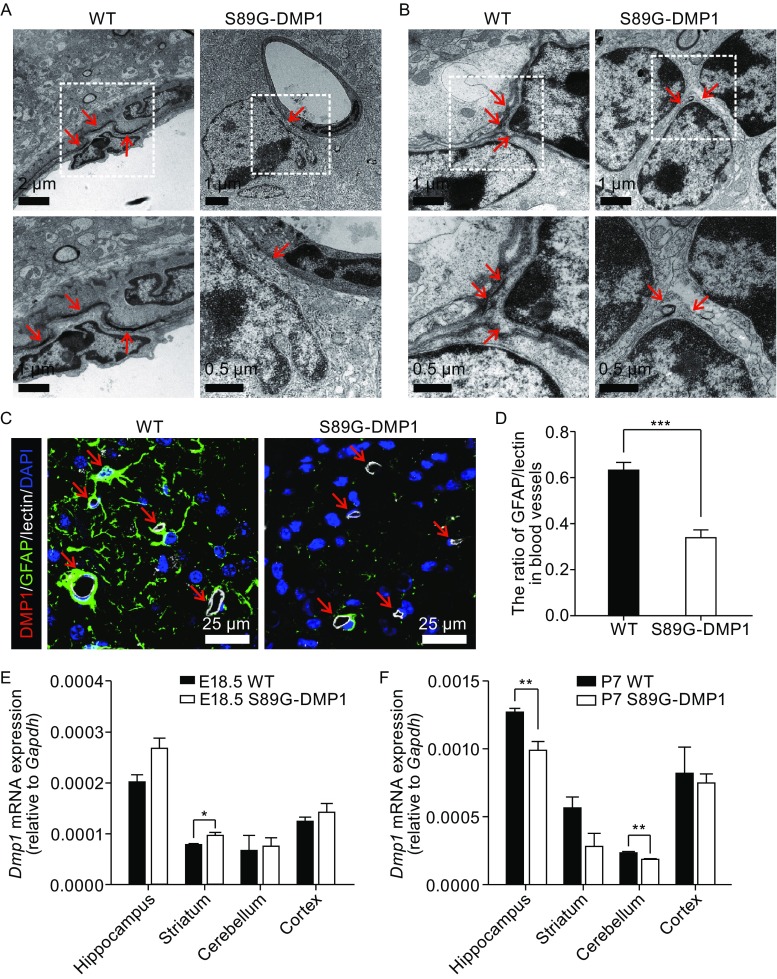
**S89G-DMP1 inhibits astrocytes to locate to and wrap around blood vessels**. (A) Transmission electron microscope showed loosened cell adhesion between astrocytes and vascular endothelial cells in the retrosplenial granular cortex (RSG) of S89G-DMP1 mice; and between astrocytes themselves (B); (C) Representative images of GFAP/lectin in the RSG, indicative of attenuated targeting of astrocytes to blood vessels in S89G mice; (D) Quantification plot for (C). ***, *P* < 0.001. At least 23 random captures from 7 mice per genotype were quantified. (E) Dmp1 mRNA decreased in different brain regions at embryonic Day 18.5 (*P* ≤ 0.05 vs. WT) and (F) at postnatal Day 7 (*P* ≤ 0.01 vs. WT). *n* = 3–4 mice per genotype

To study the ultrastructure of BBB units, we examined the retrosplenial granular cortex (RSG), where GFAP expression was abundant, from wild type and S89G-DMP1 mice using transmission electron microscope. Cell adhesions between astrocytes and endothelial cells (Fig. 2A) as well as between astrocytes and astrocytes (Fig. 2B) in S89G-DMP1 mice appeared to be disrupted based on broadened space and/or disappearance of electron-dense structures between cells (Fig. 2A and 2B). Using GFAP immunofluorescent staining together with lectin, we found that S89G-DMP1 mutant mice had less astrocyte end feet wrapping round blood vessels (Fig. 2C and 2D). We quantified the fluorescence intensity of DMP1 expression in brain and found that DMP1 protein expression level did not change (Fig. S5A and S5B). To identify whether deglycosylation has an impact on the expression of *Dmp1*, we examined the *Dmp1* mRNA expression levels by using qPCR in different brain regions. We found that the *Dmp1* mRNA expression level was significantly increased in both WT and S89G-DMP1 brain after birth (Fig. 2E and 2F). However, S89G-DMP1 mutation did not significantly change cortical *Dmp1* mRNA levels, while in other brain regions small changes might occur.

### S89G-DMP1 astrocytes manifested cell shrinkage and displayed loose cell contact

**Figure 3:**
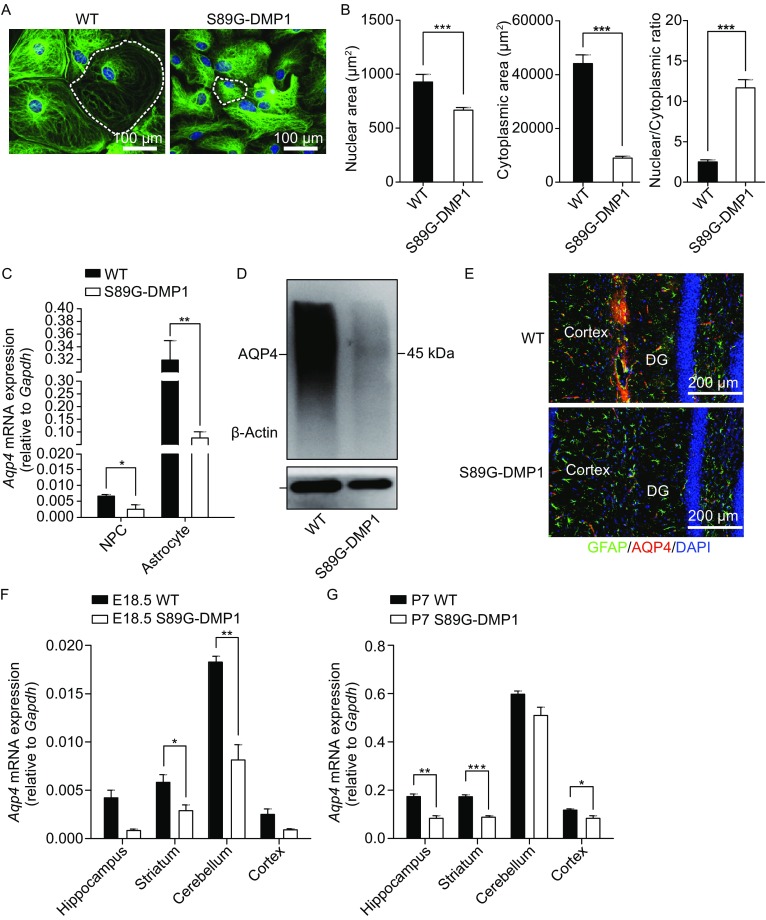

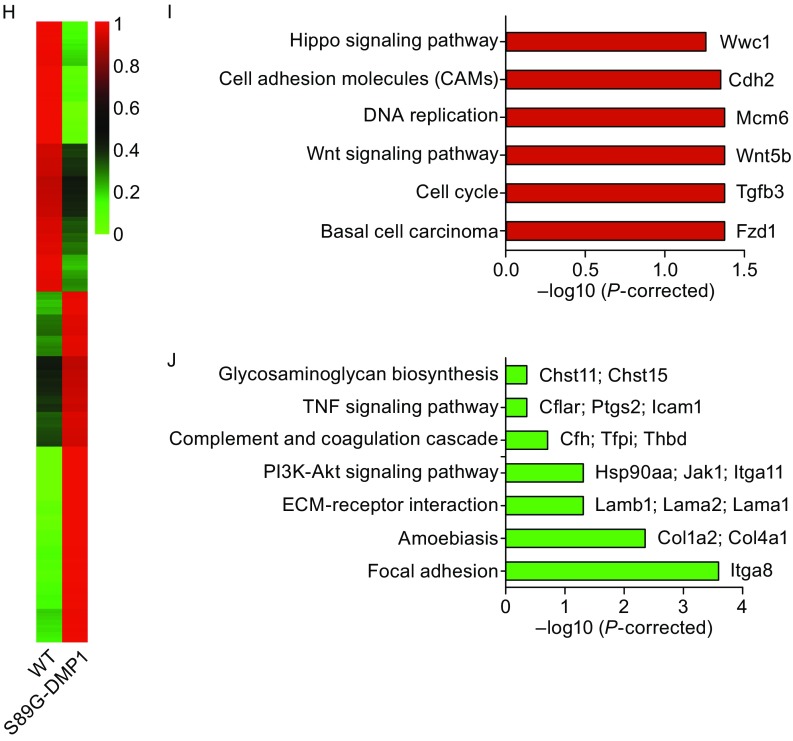
**Reduction of astrocyte volume and AQP4 expression in S89G-DMP1 astrocytes**. (A) Primary astrocytes cultures derived from S89G-DMP1 showed shrinkage in morphology; (B) The ratio of nucleus to cytoplasm decreased in S89G-DMP1 astrocytes; *n* = 32 cells for WT; *n* = 104 cells for S89G-DMP1. (C) AQP4 mRNA expression and (D) protein expression were decreased in astrocytes differentiated from NSCs; (E) Immunofluorescence staining showed decreased AQP4 protein expression in S89G-DMP1 cortex; (F) AQP4 mRNA in different brain regions of WT and S89G-DMP1 mice at embryonic Day 18.5, and (G) at postnatal Day 7; *n* = 3–4 mice per genotype. (H) Heatmap of differentially expressed genes in S89G-DMP1 astrocytes compared to WT controls; up-regulated (I) and down-regulated KEGG pathways (J) in S89G-DMP1 astrocytes

We isolated astrocytes from S89G-DMP1 adult mouse cortex and generated primary astrocyte cultures (Figs. 3A and S2). Cultures went through passaging in the presence of fetal bovine serum. The cellular composition of the subcultures was analyzed over a period of 3 weeks after immunostaining with an astrocyte marker GFAP. We observed that S89G-DMP1 astrocytes manifested cell shrinkage and displayed loose cell-cell contact (Fig. 3A and 3B), with the area of nuclei and cytoplasm significantly reduced and the ratio of nuclear to cytoplasmic areas dramatically increased (*P* ≤ 0.05 vs. WT, Fig. 3B). To clarify whether deglycosylation has an effect on the initiation of gliogenesis, we isolated and cultured neural stem cells (NSCs) from S89G-DMP1 mice and found that S89G-DMP1 NSCs demonstrated hyper proliferation *in vitro* compared to WT (Fig. S3A and S3B). Interestingly, neurospheres derived from NSCs of S89G-DMP1 mice were quite heterogeneous in size, (Fig. S4A and S4B), suggesting that different NSC clones might respond differently towards DMP1 S89G mutation. To determine whether glycosylation of DMP1 affects NSC differentiation potentials, we removed mitogens EGF and bFGF from NSC cultures to allow spontaneous differentiation. We observed that deglycosylation of DMP1 had no effect on NSC spontaneous differentiation into neurons or astrocytes (Fig. S3C and S3D). In addition, there were no differences in cell apoptosis between WT and S89G-DMP1 NSCs (Fig. S3E and S3F). These data suggested that DMP1 glycosylation was mainly involved in astrocyte maturation.

### AQP4 expression was attenuated in S89G-DMP1 astrocytes

Aquaporin-4 (AQP4) expression represents normal polarity of astrocyte at BBB. To investigate the association between BBB disruption and astrocyte phenotype, we examined changes in *Aqp4* mRNA and protein levels both *in vitro* and *in vivo*. We directly differentiated primary NSCs into astrocytes in the presence of BMP-2 and LIF, and found that AQP4 expression levels increased significantly after NSC differentiation into astrocytes. However, AQP4 expression was significantly lower both in NSCs and after their differentiation into astrocytes with S89G-DMP1 mutation (Fig. 3C and 3D). Immunofluorescence staining also showed that AQP4 protein expression decreased dramatically in cortex (Fig. 3E). Moreover, we found that *Aqp4* mRNA levels of S89G-DMP1 mice significantly decreased also in cerebellum and striatum at E18.5 and in hippocampus, striatum, and cortex at P7 (Fig. 3F and 3G). Given that AQP4 is an important element for BBB function, such a strong phenotype with AQP4 is consistent with BBB functional disruption.

### Transcriptome changes in the S89G-DMP1 NSCs and astrocytes

To obtain an overall unbiased mechanistic insight into the biological function of DMP1 glycosylation in astrocytes, we performed RNA sequencing of WT and S89G-DMP1 primary astrocyte cultures. We found significant changes in gene expression, including 991 genes being up-regulated and 762 genes being down-regulated (Fig. 3H, Table 1), which would be categorized into 13 pathways, according to Kyoto encyclopedia of genes and genomes (KEGG) analysis (Table 2). Among these pathways, molecules in the TGF-beta signaling pathway, ECM interaction and focal adhesion pathway were down-regulated (Fig. 3J), whereas Wnt signaling and cell cycle pathway were up-regulated (Fig. 3I). These results suggested that glycosylation of DMP1 was involved in cell proliferation and differentiation. Moreover, cell-cell adhesions seemed to be one of the main functions of glycosylated N-DMP1, supporting the notion that glycosylated N-DMP1 from astrocytes assure proper structure and function of BBB through organizing ECM (Laminins, integrins, collagens, and etc.), therefore acting as a key molecular regulator for BBB.

In addition, we cultured neurospheres in serum-free medium and found that the size of S89G-DMP1 neurospheres was no longer uniform as wild type ones. To further clarify whether there are changes in transcriptome as early as in NSCs, we performed transcriptome analyses. Result indicated that compared to WT, 343 genes were up-regulated and 474 genes were down-regulated in NSCs of S89G-DMP1 mice, (Fig. S4C, Table 3). According to gene ontology (GO) analysis, changes in gene expression could be categorized into 11 pathways (Table 4). Among these pathways, again, molecules in the cell adhesion (laminins, integrins, CSPGs) were down-regulated, while gene involved in cell cycles were up-regulated. These data suggested that changes in the expression of cell adhesion and cell proliferation genes occurred as early as in NSCs and maintained after NSC differentiation into astrocytes with deglycosylation of DMP1.

### DMP1 overexpression could resist the reversible opening of blood-brain barrier caused by mannitol

**Figure 4:**
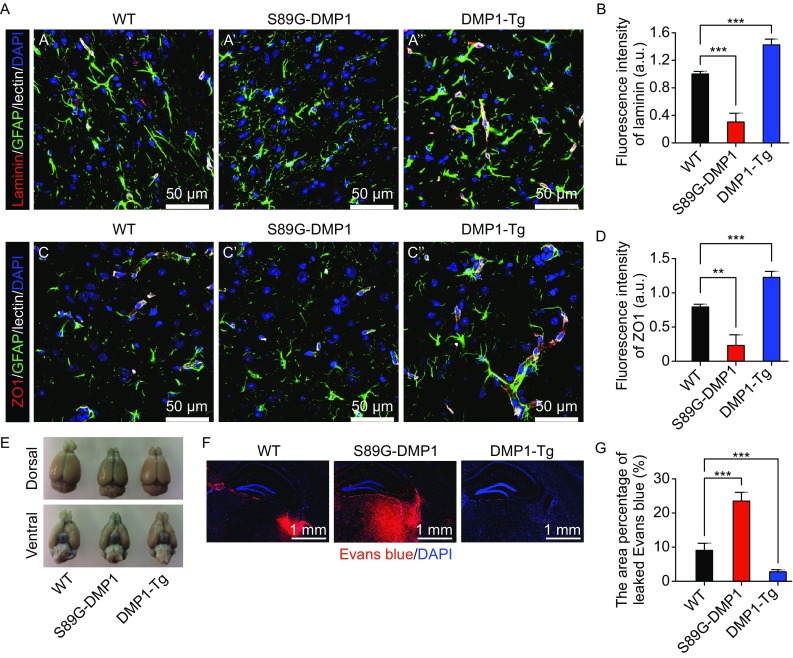
**Dmp1 overexpression could resist the reversible opening of blood-brain barrier caused by mannitol**. Mouse retrosplenial granular cortex showing expression of laminin (A) and ZO1 (C) at lectin-labeled vasculature, with GFAP immunofluorescence; (B) Quantification of (A); (D) quantification of (C); **, *P* < 0.01, ***, *P* < 0.001, mean ± SEM; a.u. represents arbitrary unit. At least 9 random captures from 3 mice per genotype were quantified. (E) Brains dissected from mice injected with Evans blue intraperitoneally immediately after tail vein injection of mannitol; (F) After perfusion, brain sections were used to examine Evans blue directly under fluorescent microscope. (G) Quantification of (F). ***, *P* < 0.001, mean ± SEM (Scale bar: 500 μm). At least 9 random captures from 3 mice per genotype were quantified

To further study the function of DMP1-PG in protection of BBB, we overexpressed DMP1 in mouse brain using a nestin promoter driver and established DMP1 overexpression transgenic mice, named DMP1-Tg (Fig. S5A and S5B). In DMP1-Tg mice, we observed that strong protective effect of DMP1-Tg on the blood-brain barrier, mainly by increasing the expression of laminin and ZO1 (Fig. 4A–D) without altering astrocyte polarity, morphology, and proliferation (Fig. S5C–F). These changes protected BBB to resist the osmotic pressure of mannitol on the opening of the blood-brain barrier.

Rapid intravenous infusion of mannitol, can cause high osmotic pressure. Under high osmotic pressure, the tight junctions of cerebral vascular endothelium can be reversibly opened, which can promote entry of some molecules into the brain tissue. The opening of the blood-brain barrier also takes a certain amount of water from the brain tissue and produces a certain degree of vasodilation, thereby increasing cerebral blood volume, and leading to cerebrovascular endothelium distraction, which leads to further opening of tight junctions between brain endothelial cells (Bhattacharjee et al., 2001; Pan et al., 2000). We performed distal intravenous administration of mannitol, and assumed that overexpression of DMP1 could resist the vasodilation. Immediately after the administration of mannitol, we intraperitoneally injected Evans blue into the wild type C57 mice, the DMP1-Tg mice and the S89G-DMP1 mice. Respectively compared to two other groups, we found that specific overexpressed DMP1 in nestin positive cells could resist BBB reversible opening induced by mannitol (Fig. 4E–G). Both gain- and loss- of function studies strongly supported important roles of astrocytic DMP1 glycosylation in maintaining the structure and function of BBB.

## DISCUSSION

The BBB is mainly composed of brain microvascular endothelium, astrocytic end feet, pericytes, and basement membrane (BM) (Persidsky et al., 2006; Alvarez et al., 2011; Zhao et al., 2015; Armulik et al., 2010). Astrocytes tightly wrapped around blood vessels and formed a unique paracellular and transcellular barrier to protect brain parenchyma from blood-borne solutes. To accomplish the sealing function, astrocytic end feet target to vessels through cell-cell adhesions (Yao, et al., 2014), which are necessary to form the impermeable blood brain barrier. However, how astrocytic protein contributes to this process remains not fully understood.

**Figure 5:**
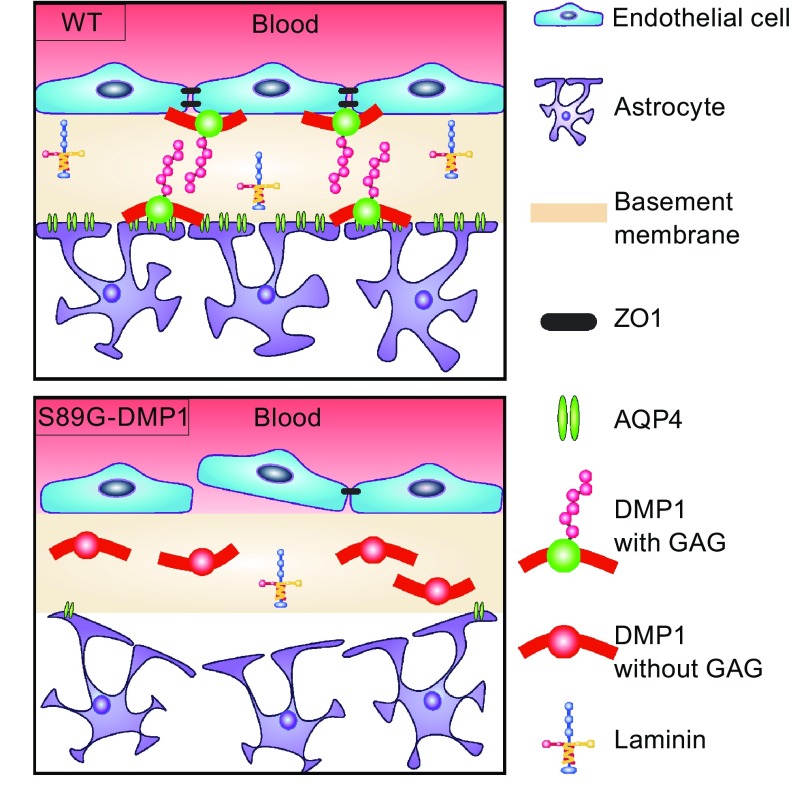
**Proposed model of how deglycosylation of DMP1 affects astrocyte maturation and BBB integrity**. Under physiological conditions, showed in WT, astrocytes could accurately target to brain blood vessels, wrap around vessels, and seal the cell gaps between endothelial cells through cell-cell interaction. When the GAG chain of DMP1 is lost during pathological state, astrocytes shrink, and signified by the reduction of AQP4 and laminin. As a result, astrocytes have attenuated their propensity of targeting to vessels, and fail to accomplish the sealing function, leading to BBB leakage

We observed remarkable phenotypic shrinkage of astrocytes resulted from a lack of DMP1 glycosylation in cellular microenvironment, which was predictive of propensity for diverse diseases, including BBB disruption-mediated brain disorders (Fig. 5). The selective removal of glycosylation of DMP1 not only led to phenotypic shrinkage and cell polarity vanishing of astrocytes at BBB, but also caused hyper proliferation of NSCs and astrocytes. More importantly, we had discovered dramatic disruption of BBB in S89G-DMP1 mice. Conversely specific overexpression of DMP1 in mice was able to resist mannitol-induced BBB reversible opening. Both gain- and loss-of function studies strongly supported an essential role of DMP1 glycosylation in regulating BBB function.

Transcription analyses systemically, and in an unbiased manner, revealed that the main functions of DMP1 glycosylation was to sustain expression of ECM components including laminins, collagens, integrins, and CSPGs from astrocytes and/or NSCs, and at the same time suppressed cell proliferation, thus allowing astrocytes to stay in a more differentiated functional state. This could be the main underlying molecular mechanism by which DMP1 glycosylation regulated BBB structure and function.

It was quite surprising to discover that a bone and dentine mineralization regulator DMP1 was involved in regulating BBB. DMP1 is a molecule with two parts. The full length DMP1 is almost always cleaved into N-terminal and C-terminal moieties, thus full length DMP1 is hardly detectable (Qin, et al., 2006). The C-terminal moiety is known to play critical roles in regulating bone and dentine development, while the N-terminal facilitates osteogenesis (Sun, et al., 2015). Interestingly, the C-terminal moiety of DMP1 was not involved in BBB function because C-DMP1 knockout did not show BBB disruption. Glycosylation of the N-terminal DMP1, on the other hand, was crucial for regulation of BBB structure and function. This indicated that functional diversity of a single gene or protein could emerge from domain segregation as well as post-translational modifications of proteins.

BBB disruption has been associated with many neurological disorders, including Alzheimer’s disease (AD), amyotrophic lateral sclerosis, Parkinson’s and Huntington’s diseases (Sweeney et al.,^2015^; Winkler et al. 2013; Korczyn, 2015; Drouin-Ouellet et al., 2015). Using AD as an example, it is known that 70%–85% of amyloid-β (Aβ), a major component of AD pathology, is cleared from the brain through trans-vascular clearance across the BBB (Tarasoff-conway, et al., 2015). *APOE4*, an AD risk allele, is found to not only stimulate Aβ production, but also damages BBB and impairs the clearance of Aβ. (Deane et al., 2008; Huang et al., 2017). In human *APOE4*-expressing mice BBB breakdown precede neuronal dysfunction and behavioral deficits (Bell et al., 2012). Moreover, *APOE4* is found to increase BBB damage in AD patients (Zipser et al., 2007; Halliday et al., 2016). These evidences strongly indicate that BBB disruption is critically involved in the onset and progression of neurodegeneration, therefore finding key molecular regulators for BBB may open up a new path towards developing therapies to treat a number of neurological disorders. Taken together, our study not only revealed a novel function of DMP1 but also uncovered a novel factor regulating BBB structure and function, which would have potential therapeutic values.

## MATERIALS AND METHODS

### Animals

S89G-DMP1 knock-in point mutation mouse model was created with homologous recombination method by Beijing Biocytogen Co., Ltd, China. In brief, the S89G mutation was introduced in exon 6 using an overlap extension-PCR method. Homology regions covering 6.6 kb upstream of Dmp1 exon 6 and 8.2 kb downstream of exon 6 were subcloned and FRT-flanked Neo resistance positive selection cassette was inserted into 179 bp downstream of exon 6. The targeting vector was transfected into C57BL/6J embryonic stem (ES) cells by electroporation. Eight positive clones were identified by Southern blotting with 5′ probe and 3′ probe. Three positive clones were injected into Balb/c blastocysts and implanted into pseudopregnant females. Five chimeric male mice were crossed with C57BL/6J females to obtain F1 mice carrying the recombined allele containing the S89G mutation and Neo selection cassette. The presence of the S89G mutation was further verified by sequencing. Heterozygous males were mated with B6.129S4-Gt (ROSA) 26-Sortm1 (FLP1) Dym/RainJ females (Jackson Laboratories), to remove the NEO cassette. Homozygous mutant mice were obtained by intercrossing the heterozygous littermates. Eight positive clones were selected, 3 pups were collected in first litter. Genotyping of S89G-DMP1 mouse primers used for S89G point mutation identification: Dmp1-Neo-F-primer, CGCATTGTCTGAGTAGGTGTC; R-primer, GGTTCTTACATGGGCAGGATAAGC. Transgene PCR product size: 306 bp.

DMP1-Tg mouse model was created with Gateway, a bacteriophage-based homologous recombination method by Cyagen Bioscience Inc. (Guangzhou, China). Briefly, we microinjected pRP.ExBi-nestin-Dmp1-IRES-eGFP DNA into mouse zygote to overexpress *Dmp1* with nestin promoter. Homozygous transgenic mice were obtained by inter-crossing heterozygous littermates with C57BL/6 background. Two male pups and 4 female pups were collected in first litter. Genotyping of DMP1 mouse-primers used for DMP1-Tg mouse identification: transgene PCR primer F1, AACTTTCCCCGGAGCATCCAC; transgene PCR primer R1, TCTGTACTGGCCTCTGTCGTA; internal control PCR primer F, CAACCACTTACAAGAGACCCGTA; internal control PCR primer R, GAGCCCTTAGAAATAACGTTCACC. Transgene PCR product size: 351 bp; internal control PCR product size: 632 bp.

Wild-type C57BL/6 mice were used as control. Pregnant mice were obtained following overnight mating (Day of vaginal plug was defined as embryonic day 0.5). All animals were raised in a specific pathogen-free (SPF) facility, under a 12 h:12 h day–night illumination cycle. For sacrificing mice, animals were killed by cervical dislocation after routine anesthesia. The animal care and use procedures were approved by the Animal Welfare Committee of School of Stomatology, Tongji University (Shanghai, China).

### Immunofluorescence

For immunofluorescence assay, mice received routine anesthesia were successively perfused with PBS and followed by 4% (*w*/*v*) paraformaldehyde (PFA). And then, tissues were post-fixed with 4% PFA at 4°C overnight, cryopreserved in 30% sucrose and frozen in TissueTek OCT (Sakura), and sectioned at a thickness of 10 μm. Tissue sections were permeabilized with 0.1% Triton X-100 in PBS and blocked with 3% donkey serum in PBS. For immunocytochemistry, coverslips plated with cells were washed with PBS twice and fixed with 4% PFA at room temperature for 20 min. After this, coverslips were permeabilized with 0.3% Triton X-100, blocked with 3% donkey serum, and processed to staining procedures. After incubation with the following primary antibodies, anti-N-DMP1 (1:200; monoclonal anti-N-DMP1 9B6.3 antibody, reported previously by Qin et al., 2006), anti-Tuj1 (1:500; R&D, MAB1195), anti-laminin (1:500; abcam, ab7463), anti-ZO1 (1:100; Invitrogen, 40-2200), anti-Ki67 (1:500; abcam, ab15580), anti-GFAP (1:1000; Dako, Z0334), anti-GFAP (1:1000; cell signaling), anti-AQP4 (1:500; Millipore, AB3594), and anti-BrdU (1:300; abcam, ab6326) at 4°C overnight, samples were then incubated with 647/568/488 Alexa Fluor-conjugated secondary antibodies (1:500, Invitrogen) and/or with lectin-FITC (Dylight, DL-1174) for 2 h at room temperature. Finally, slides were mounted in Fluoromount G (SouthernBiotech, 0100-01) and visualized by fluorescence, or confocal microscopy.

### Blood-brain barrier permeability assay

Control and transgenic mice were injected intraperitoneally with 2% Evans blue in saline at the concentration of 10 μl/g (of body weight) twelve hours before the mice were killed. Animals were perfused with saline before brains were collected and sectioned. BBB breakdown was revealed by visualizing Evans blue under the fluorescence microscope.

### Cell culture

Primary mouse astrocytes were isolated and cultured as described: mouse cortex from 3-month-old WT and S89G-DMP1 mice (both genders) were collected under aseptic conditions, followed by digestion with 2% papain in HBSS. After centrifugation, the pellet was re-suspended in HBSS and centrifuged at 200 ×*g* for 5 min. These vessels were sequentially filtered through 100-µm and 40-µm cell strainers. The single cells were plated into pre-coated plate, and then grown in DMEM supplemented with 10% FBS.

### RT-PCR

Total RNA was extracted from dissected tissues or cultured cells using Trizol reagent (Invitrogen, 15596-018), according to the manufacturer’s instructions. RNA was further purified by DNAse treatment and removal kit (Ambion, AM1906). Equal amount of total RNA was subjected to reverse transcription using SuperScript III First-Strand Synthesis kit (Invitrogen, 18080-051) as instructed. Real-time PCR was performed on a 7500 or Q7 real-time PCR system (Applied Biosystems) by using SYBR Premix Ex Taq with ROX (Takara, RR820B). For real-time PCR, primers used for gene expression are listed below: Dmp1, GGTGATTTGGCTGGGTC, TGTGGTCACTATTTGCCTGT; Gapdh, CCTCGTCCCGTAGACAAAATG, TCTCCACTTTGCCACTGCAA, Aqp4, TTTGGACCCGCAGTTAT, AAGGCGACGTTTGAGC;

The mRNA expression level was normalized to housekeeping gene GAPDH. Results are shown as mean ± SEM.

### Chondrointinase ABC (ChABC) treatment

Acidic proteins including DMP1 were extracted from whole brain and applied to a Q-Sepharose column to purify the DMP1 components by using a gradient of NaCl (0.1 to 1.2 mol/L). Protein samples were reconstituted with reaction buffer containing 50 mmol/L Tris-HCl, 60 mmol/L sodium acetate, pH = 8.0, 0.02% BSA; add 0.01 unit (1 µL) of chondrointinase ABC (sigma, C2905) per 30 µg protein. Incubate all the samples at 37°C for 2 h, dry the samples by SpeedVac and then reconstituted for SDS-PAGE.

### Western blot

Total protein concentration from the lysates was determined using the Bio-Rad protein assay kit. Equal amount of protein was loaded and separated in 10% or 12% SDS-PAGE, followed by transfer to PVDF membrane (Millipore). The membranes were then incubated with anti-N-DMP1 (1:1000; monoclonal anti-N-DMP1 9B6.3 antibody), anti-AQP4 (1:1000; Millipore, AB3594), anti-beta actin (1:2000; abcam, ab8227), anti-GAPDH (1:5000; abcam, ab128915) at 4°C overnight, followed by incubation with HRP-conjugated secondary antibodies (1:5000; Jackson Lab, 111-035-003 or 115-035-003). The proteins were visualized by Immobilon Western Chemiluminescent HRP Substrate (Millipore, WBKLS0100).

### The transmission electron microscope

3-month-old mice (both genders) were anaesthetized and perfused with PBS followed by physiological saline buffer containing 2% PFA and 2% glutaraldehyde. After perfusion, the cortex was dissected out and post-fixed with 2% glutaraldehyde overnight. Tissues were washed three times with phosphate buffer and post-fixed in 1% osmic acid, and then were washed three times with phosphate buffer and dehydrated with acetone. Tissues were placed in the EPON for 2 h and then incubated with pure resin at 68°C for 48 h to complete the polymerization. Next, tissues were cut into ultra-thin sections and stained with uranyl acetate and lead citrate for capture.

### RNA sequencing data analysis

The establishment of cDNA library and RNA sequencing were performed by Novogene Co., Ltd., Beijing. Raw data (raw reads) of fastq format were firstly processed through in-house perl scripts. In this step, clean data (clean reads) were obtained by removing reads containing adapter, reads containing ploy-N and low-quality reads from raw data. All the downstream analyses were based on the clean data with high quality. Index of the reference genome was built using Bowtie v2.0.6 and paired-end clean reads were aligned to the reference genome using TopHat v2.0.9. RPKM of each gene was calculated based on the length of the gene and reads count mapped to this gene, considering the effect of sequencing depth and gene length for the reads count at the same time. All the differentially expressed genes were used for heatmap analysis and Kyoto encyclopedia of genes and genomes (KEGG) analysis. For KEGG analysis, a Q-value < 0.05 was used as the threshold to determine significant enrichment of the gene sets. RNA-seq data was deposited at GSE99784.

### Statistical analysis

Data were presented as mean ± SEM, and statistical differences were determined with unpaired Student’s *t*-test via SPSS 20.0.

## Electronic Supplementary Material

Below is the link to the electronic supplementary material.

Supplementary material 1 (PDF 733 kb)

Supplementary material 2 (PDF 76 kb)

Supplementary material 3 (XLSX 75 kb)

Supplementary material 4 (XLSX 10 kb)

Supplementary material 5 (XLSX 21 kb)

Supplementary material 6 (XLSX 11 kb)
