# Correction to: Glycosylation of dentin matrix protein 1 is a novel key element for astrocyte maturation and BBB integrity

**DOI:** 10.1007/s13238-018-0574-z

**Published:** 2018-08-29

**Authors:** Bo Jing, Chunxue Zhang, Xianjun Liu, Liqiang Zhou, Jiping Liu, Yinan Yao, Juehua Yu, Yuteng Weng, Min Pan, Jie Liu, Zuolin Wang, Yao Sun, Yi Eve Sun

**Affiliations:** 1 Tongji University School of Medicine, Stem Cell Translational Research Center, Tongji Hospital, Shanghai, 200065 China; 2 Department of Oral Implantology, School of Stomatology, Tongji University, Shanghai Engineering Research Center of Tooth Restoration and Regeneration, Shanghai, 200072 China; 3 Department of Psychiatry and Biobehavioral Sciences, David Geffen School of Medicine, University of California, Los Angeles, CA 90095 USA; 4 Collaborative Innovation Center for Brain Science, Tongji University, Shanghai, 200092 China

## Correction to: Protein Cell 2018, 9(3):298–309 10.1007/s13238-017-0449-8

In the original publication, the label of Fig. 2C should be read as “GFAP/lectin/DAPI” not “DMP1/GFAP/lectin/DAPI”.

**Figure 2:**
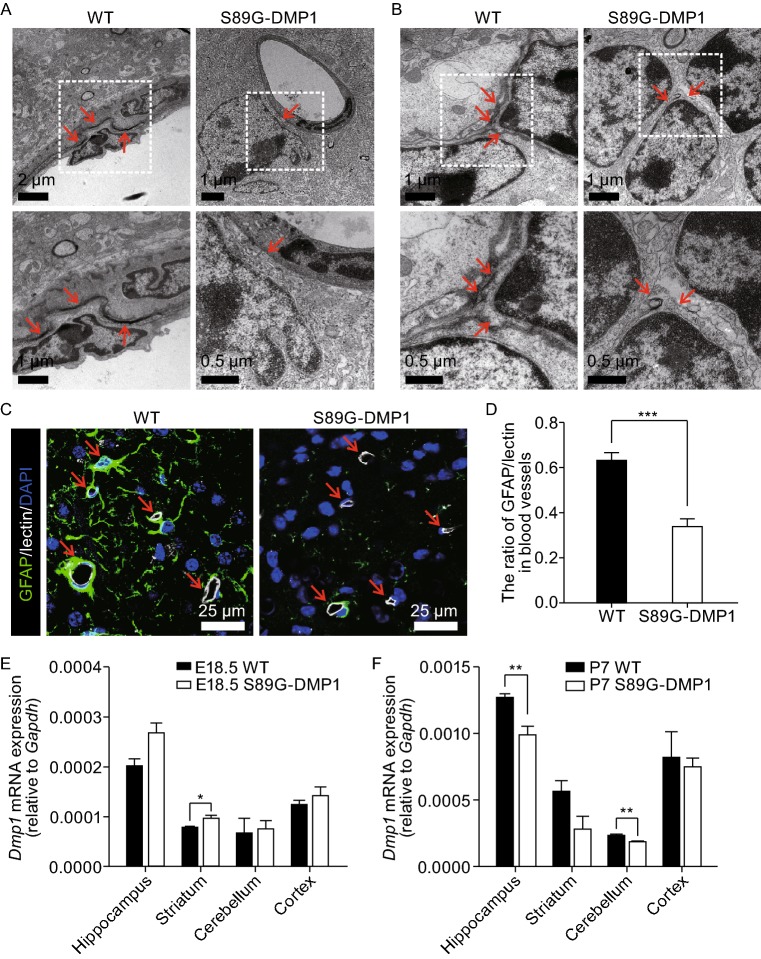
**S89G-DMP1 inhibits astrocytes to locate to and wrap around blood vessels**. (A) Transmission electron microscope showed loosened cell adhesion between astrocytes and vascular endothelial cells in the retrosplenial granular cortex (RSG) of S89G-DMP1 mice; and between astrocytes themselves (B); (C) Representative images of GFAP/lectin in the RSG, indicative of attenuated targeting of astrocytes to blood vessels in S89G mice; (D) Quantification plot for (C). ***, *P* < 0.001. At least 23 random captures from 7 mice per genotype were quantified. (E) Dmp1 mRNA decreased in different brain regions at embryonic Day 18.5 (*P* ≤ 0.05 vs. WT) and (F) at postnatal Day 7 (*P* ≤ 0.01 vs. WT). *n* = 3–4 mice per genotype

