# Supplementary material for: Glycosylation of dentin matrix protein 1 is a novel key element for astrocyte maturation and BBB integrity
Source: Protein Cell. 2017 Aug 18;9(3):298–309. doi: 10.1007/s13238-017-0449-8 (PMC5829272; doi:10.1007/s13238-017-0449-8)
Supplement: Supplementary file 1 — Supplementary material 1 (PDF 733 kb) [file 13238_2017_449_MOESM1_ESM.pdf]

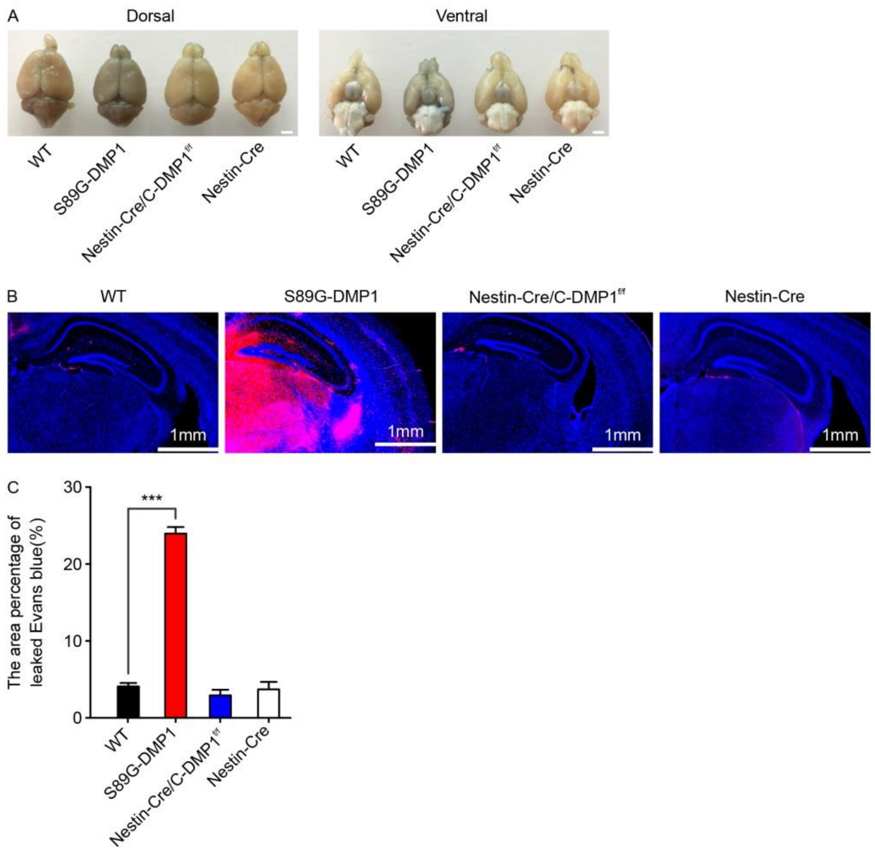

Sup. F1 Jing *et al.*

Fig. S1. C-DMP1 does not affect BBB integrity. (A) Brains dissected from mice injected with Evans blue intraperitoneally; (B) After perfusion, brain sections were used to examine Evans blue directly under fluorescent microscope. (C) Quantification of B ***, $P < 0.001$, Mean ± SEM. At least 9 random captures from 3 mice per genotype were quantified.

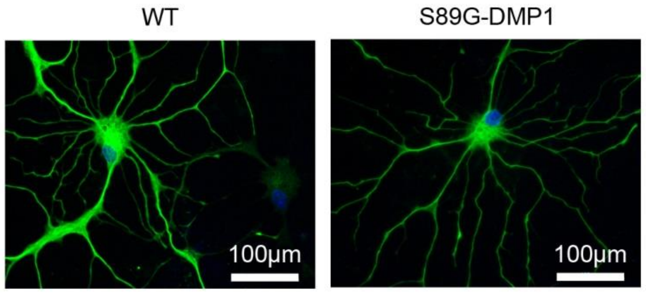

Sup. F2 Jing *et al.*

Fig. S2. The morphology of cultured astrocytes from wild type and S89G-DMP1 mice.

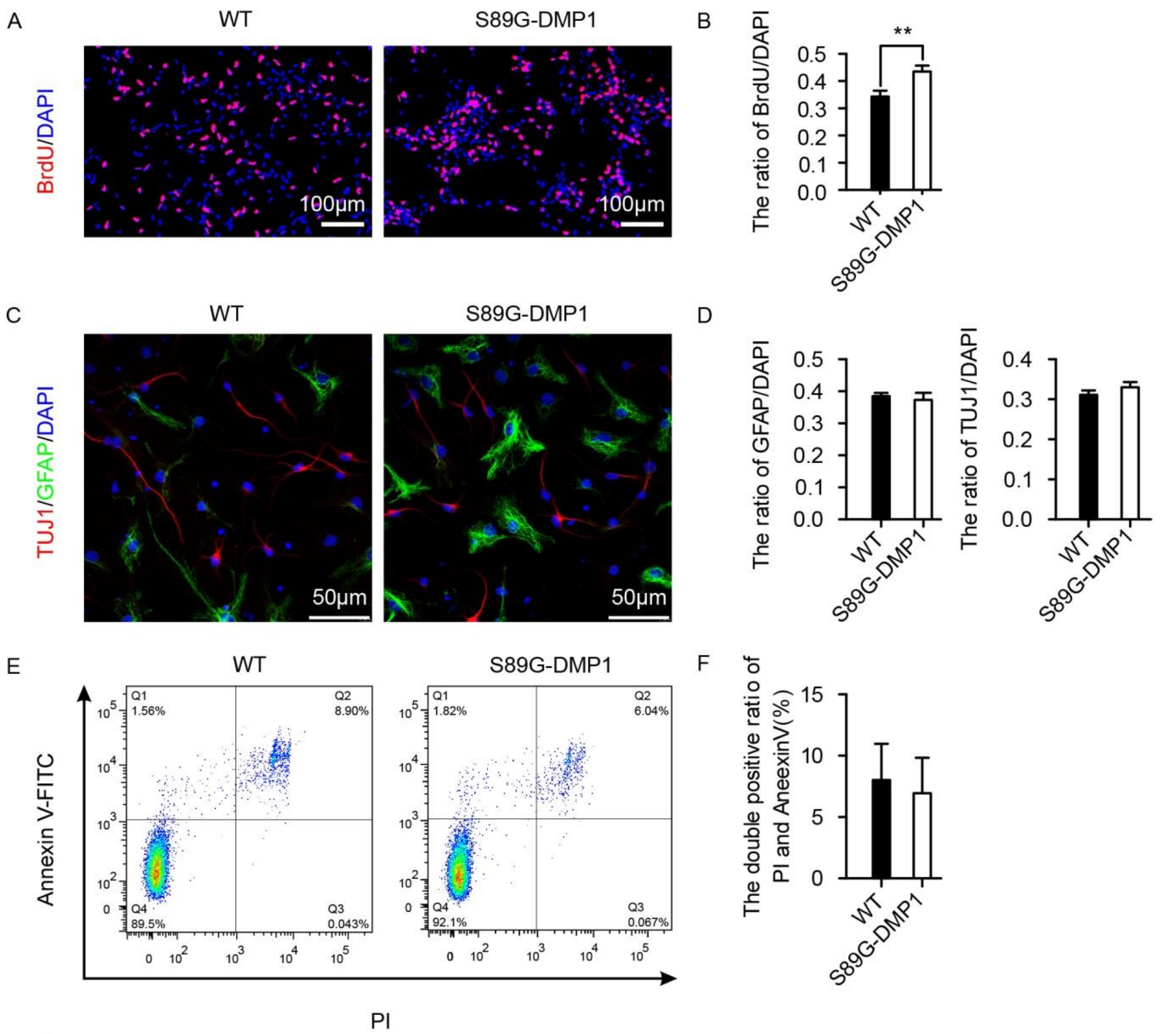

Sup. F3 Jing *et al.*

Fig. S3. Point mutation of DMP1 glycosylation site led to hyper proliferation of NSCs. (A) The BrdU immunostaining of cultured NSCs; (B) Quantification of A; 13-16 captures per genotype were quantified. (C) TUJ1/GFAP immunostaining of NSCs; (D) Quantification of C; 16 captures per genotype were quantified. (E) The flow cytometry analysis in primary NSCs using PI and Annexin; (F) quantification of E. n=4. Thus, S89G-DMP1 does not influence cell survival.

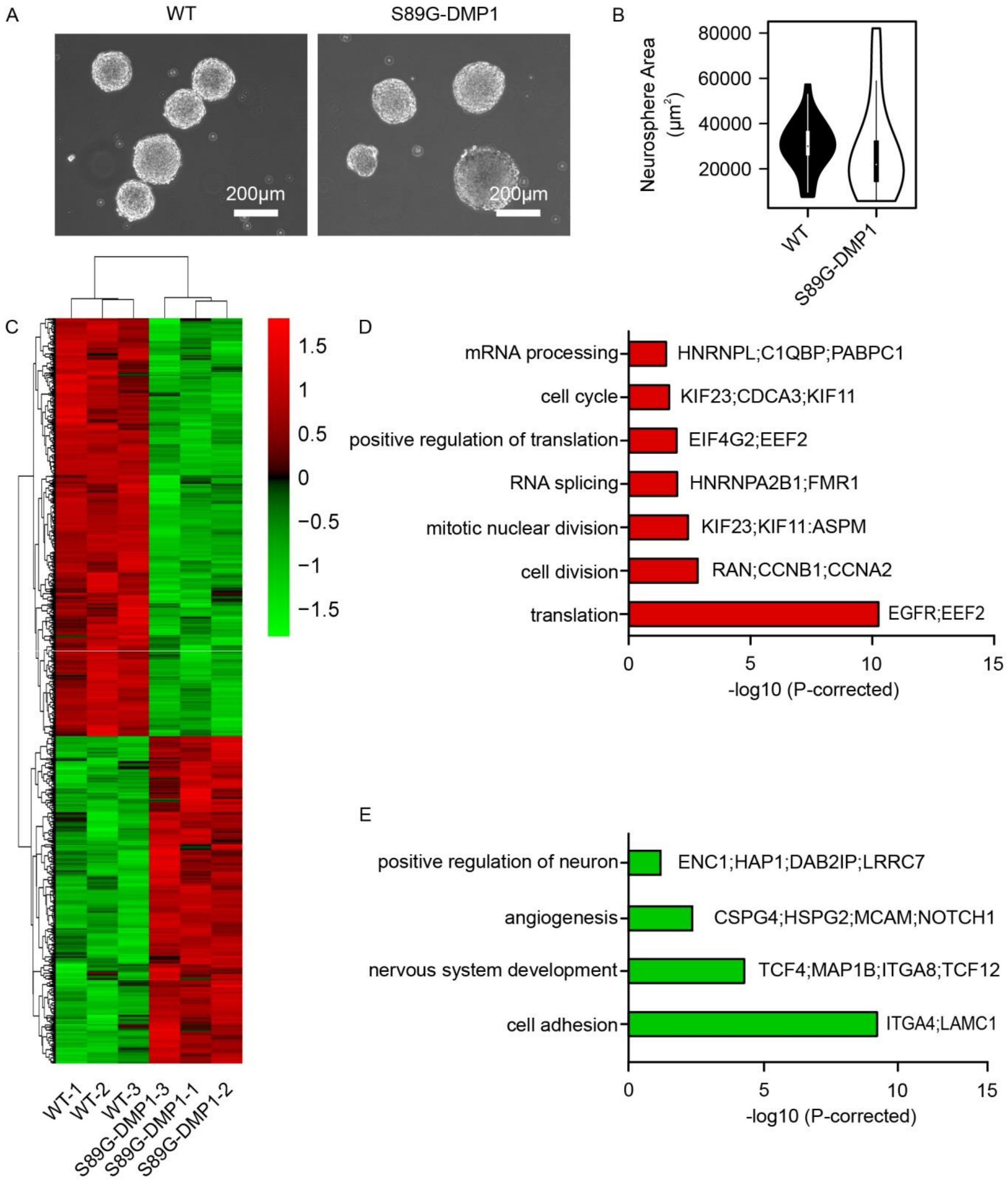

Sup. F4 Jing *et al.*

Fig. S4. The differential genes analysis between WT and S89G-DMP1 NSCs. (A) Non-homogeneous neurospheres formed from S89G-DMP1 NSCs; (B) Violin plot representing size differences of neurospheres between WT and S89G-DMP1. n=21-25 per genotype. The width and length of polygons represent the density and the range of data respectively. The thick bar indicates the range between 0.25 and 0.75 quantiles, the thin line extended from it represents the 95% confidence intervals, and the dot is the median. (C) Heatmap of differentially expressed genes between WT and S89G-DMP1 neurospheres; GO

pathways of upregulated (D) and downregulated (E) genes in S89G-DMP1 NSCs as compared to wild type.

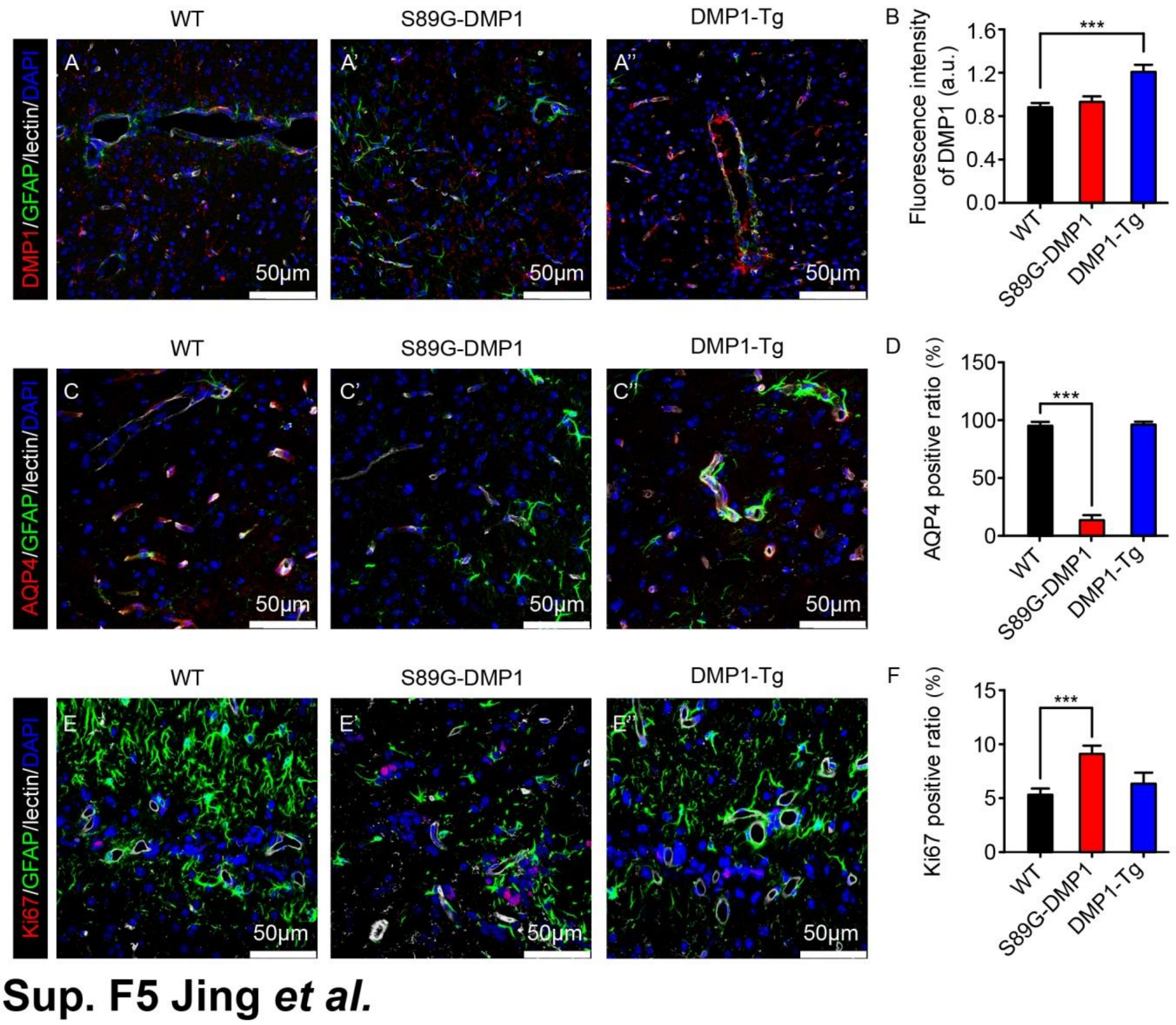

Fig. S5. Enhanced proliferation and decreased AQP4 expression in S89G-DMP1 mice, and reversed phenotype by DMP1-Tg. (A) Representative images showing expression of DMP1 in mouse retrosplenial granular cortex; (B) Quantification for A; ***, $P < 0.001$, Mean ± SEM. Representative images showing expression of AQP4(C) and Ki67(E) in mouse retrosplenial granular cortex; (D) (F) Quantification for C and E; ***, $P < 0.001$, Mean ± SEM. At least 9 random captures from 3 mice per genotype were quantified.

Table Legends

Table 1 The differential genes of S89G-DMP1 adult astrocytes revealed via RNA sequencing

Table 2 The list of KEGG pathways involved in S89G-DMP1 astrocyte differential genes.

Table 3 The differential genes of S89G-DMP1 adult NSCs revealed by RNA sequencing

Table 4 The list of GO pathways involved in differential genes in S89G-DMP1 NSCs.
