# Supplementary material for: Glycosylation of dentin matrix protein 1 is a novel key element for astrocyte maturation and BBB integrity
Source: Protein Cell. 2017 Aug 18;9(3):298–309. doi: 10.1007/s13238-017-0449-8 (PMC5829272; doi:10.1007/s13238-017-0449-8)
Supplement: Supplementary file 2 — Supplementary material 2 (PDF 76 kb) [file 13238_2017_449_MOESM2_ESM.pdf]

**Supplementary materials**

## MATERIALS AND METHODS

### Animals

The C-Dmp1 $^{flox/flox}$ mice were generated using gene targeting to replace exon 6 with loxP and neo (neomycin phosphotransferase gene) cassette as reported (Feng et al., 2006). We intercrossed Nestin-Cre mice and the C-Dmp1$^{flox/flox}$ mice to generate a truncated Dmp1 transgenic mice, which conditionally knockout C-DMP1 protein in nestin positive cells. Wild-type C57BL/6 mice were used as control. The animal care and use procedures were approved by the Animal Welfare Committee of School of Stomatology, Tongji University (Shanghai, China).

### Cell culture

Neural stem cells were isolated and cultured as described: subventricle zone in cortex from 3-month-old WT and S89G-DMP1 mice (both genders) were collected under aseptic conditions, followed by digestion with 2% papain in HBSS. After centrifugation, the pellet was re-suspended in HBSS and centrifuged at 200 g for 5 min. These vessels were sequentially filtered through 100-μm and 40-μm cell strainers. The single cells were then cultured in DMEM/F12 supplemented with 1% N2, 10 ng/ml bFGF/EGF daily additive.

### Cell apoptosis assay

Cell death was examined using the PI/Annexin V kit (Beijing zoman biotechnology, ZP327-1), according to the manufacturer's instruction. Briefly, cells were fixed in 4% PFA and permeabilized on ice, followed by incubation with PI/Annexin V antibodies mixture for 15 min at room temperature. After washing, single cell suspensions were examined by fluorescent activated cell sorting (FACS).

### RNA sequencing data analysis

The sequencing raw data was filtered to remove low-quality tags, empty reads, and reads with only one copy number. 50 base-pair paired-end reads were aligned to GRCm38 (mm10) using the Ensembl annotation gtf file version 84. Transcriptome alignment was performed using HISAT2. Unique mapped reads instead of genome mapped reads were used in further analysis. Differential expression analysis was performed with DESeq2 using raw read counts. All the differentially expressed genes were used for heatmap analysis and Gene Ontology (GO) analysis. For GO analysis, a Q-value $< 0.05$ was used as the threshold to determine significant enrichment of the gene sets. RNA-seq data was deposited at GSE99784.
